# Efficacy, Buffering Capacity and Dynamics of Saliva-Stimulating Products

**DOI:** 10.3290/j.ohpd.c_2850

**Published:** 2026-10-21

**Authors:** Heba Ashi, Guglielmo Campus, Anna Lehrkinder, Ali Alshabeeb, Peter Lingström

**Affiliations:** a Heba Ashi Associate Professor, Department of Dental Public Health, Faculty of Dentistry, King Abdulaziz University, Jeddah, Saudi Arabia. Experimental design, performed the experiments, wrote the manuscript.; b Guglielmo Campus Senior Lecturer, Department of Cariology, Institute of Odontology, Sahlgrenska Academy, University of Gothenburg, Box 450, SE-405 30 Gothenburg, Sweden; Department of Oral and Maxillofacial Sciences, Sapienza University of Rome, Italy. Idea contribution, performed the statistical evaluation, wrote the manuscript and contributed substantially to the discussion.; c Anna Lehrkinder Biomedical Scientist, Department of Cariology, Institute of Odontology, Sahlgrenska Academy, University of Gothenburg, Box 450, SE-405 30 Gothenburg, Sweden. Experimental design, performed the experiments, proofread the manuscript, performed a certain test, contributed substantially to the discussion.; d Ali Alshabeeb Biomedical Scientist, Department of Cariology, Institute of Odontology, Sahlgrenska Academy, University of Gothenburg, Box 450, SE-405 30 Gothenburg, Sweden. Performed the experiments, performed a certain test, and proofread the manuscript.; e Peter Lingström Professor, Department of Cariology, Institute of Odontology, Sahlgrenska Academy, University of Gothenburg, Box 450, SE-405 30 Gothenburg, Sweden. Idea, hypothesis, experimental design, wrote the manuscript, proofread the manuscript and contributed substantially to the discussion.

**Keywords:** buffering capacity, comparative analysis, lozenges, parotids, saliva-stimulating products, salivary flow rate, temporal dynamics, xerostomia

## Abstract

**Background:**

Saliva maintains the health and integrity of the oral cavity while composing a complex symphony within it. Saliva’s lubricating qualities not only make talking and swallowing easier, but also promote digestion by breaking down lipids and carbohydrates through enzymatic processes.

**Aim:**

To compare the efficacy, buffering capacity and temporal dynamics of six different saliva-stimulating products in a randomised controlled crossover trial.

**Methods and Materials:**

Twelve healthy adults were enrolled, each testing six products (chewing gum with mint, Salivin, Jaw Peer medium, Jaw Peer large, Jaw Peer small and control) on separate days. Stimulated saliva secretion rate, buffering capacity, and pH were measured at baseline and at 1, 3 and 6 min following product use. Comparison between products and different time points for the same product was performed using two-way ANOVA followed by Tukey’s post hoc test. A significance level of *p* < 0.05 was applied for statistical significance.

**Results:**

Chewing gum with mint and Jaw Peer large produced the highest stimulated saliva secretion rates. The Jaw Peer product showed the greatest decrease in buffering capacity, while Salivin showed stable buffering capacity over time. Significant temporal variations in buffering capacity were observed for all products. No significant differences were found in unstimulated salivary secretion rate between products.

**Conclusions:**

Unstimulated salivary flow rate products exhibit different effects on salivary flow, buffering capacity, and temporal dynamics. Choice of product should be individualised based on patient preferences, specific clinical needs and duration of action, although very limited improvement was noticed with these saliva-stimulating products compared to chewing paraffin alone.

Saliva orchestrates a multi-faceted symphony within the oral cavity and safeguards its health and integrity.^14^ Beyond facilitating speech and swallowing through lubricating properties, saliva aids in digestion via enzymatic degradation of carbohydrates and lipids.^14^ More crucially, it boasts robust antimicrobial defences, wielding for example lysozyme, lactoperoxidase, and defensins as weapons against pathogenic bacteria and fungi.^9^ However, hyposalivation, a chronic and debilitating condition characterised by a significant reduction in salivary flow, disrupts this vital symphony, jeopardising oral health and posing a substantial threat to well-being.^1^ Notably, saliva’s buffering capacity serves as a potent shield against the acidic onslaught of oral bacteria, neutralising harmful metabolites and preventing demineralisation and enamel erosion.^4^


Management of dry mouth needs a multi-pronged approach, encompassing strategies like enhanced hydration, parotid gland stimulation techniques, and the strategic deployment of saliva-stimulating products. Traditionally, sugar-free chewing gums have reigned supreme in this domain, leveraging their mechanical action to stimulate particularly parotid gland activity and augment salivary flow.^13^ The landscape of therapeutic options has blossomed in recent years, with lozenges, sprays, electrical stimulators, and an array of novel products now vying for dominance in the quest to alleviate the discomfort and oral health complications associated with xerostomia.^6^


Despite this burgeoning selection, a critical knowledge gap persists. Clinicians and patients alike navigate the complex terrain of saliva-stimulating products with considerable uncertainty, lacking robust comparative data to guide informed decision-making. To bridge this gap, the present study embarks on a comprehensive comparative analysis of six saliva-stimulating products, encompassing both established and emerging choices. This in-depth investigation delves into three key parameters – efficacy, buffering capacity and temporal dynamics – offering vital insights to clinicians.

Quantifying the efficacy, i.e. the ability of each product to stimulate salivary flow, is paramount. Meticulous investigation will dissect salivary flow rate both during and after product use, employing standardised protocols and validated measurement techniques. By delving beyond peak flow rates, the present study will also explore temporal dynamics, elucidating the duration of action and potential redosing requirements for each product. This granular analysis will empower clinicians to tailor therapeutic regimens, optimising salivary output and maximising patient comfort.

The ability of saliva to neutralise acidic challenges via its buffering capacity is integral to maintaining oral health. This study will rigorously assess the buffering capacity of stimulated saliva from each product, employing standardised pH measurements and controlled acid challenges.

The present study will not only analyse the initial buffering potential but also investigate temporal variations, revealing how effectively each product sustains its protective shield against acidic threats. This knowledge will be crucial in choosing products suitable for patients with particularly pronounced acidity issues.

The efficacy and buffering capacity of saliva-stimulating products may vary over time, influencing their clinical usefulness. Therefore, understanding both the magnitude and duration of salivary stimulation is essential for optimising product selection and redosing intervals.

Starting from this premise, a prospective comparative pilot study was planned and carried out with the aim of evaluating six prominent saliva-stimulating products in the management of xerostomia, focusing on their efficacy, buffering capacity and temporal dynamics. After assessing the above parameters by collecting resting saliva and stimulated saliva during and after use of the products, as well as the buffer capacity of stimulated saliva, we hope to provide clinically relevant data that can guide individualised management strategies for patients with xerostomia.

## Methods and Materials

### Study Design and Participants

A total of 12 participants (6 women and 6 men) were recruited from among the personnel and students at the Institute of Odontology, University of Gothenburg (Sweden). Volunteers were identified through in-house advertisements. Inclusion criteria were aged between 18–25 years, overall good health with no current medication use, non-smokers, and a stimulated salivary secretion rate > 1.0 ml/min. Participation was voluntary, and individuals were informed in writing and verbally about the nature and purpose of the study before providing informed consent. The study was ethically approved by the Swedish Ethical Review Authority (Dnr 2024-08743-01).

### Test Products

The following five test products and one control condition, all market-available, were selected and used in the study.

1.Salivin (ACO Hud Nordic, Stockholm, Sweden). Salivin is a sugar-free product recommended for subjects with dry mouth. It contains fluoride and xylitol, which stimulate saliva production while being gentle on the teeth. It can be used by adults and children from 12 years of age (fluoride: 0.75 mg/tablet).2.Chewing Gum Mint Extra (Wrigley Company, Chicago, IL, USA).3.JAW PEER products (Jaw Peer small, medium and large, Vallentuna, Sweden) are made of a silicone-based polymer tested by RISE (Research Institutes of Sweden) and do not release residual products when chewed. Three different sizes were tested: small, medium, and large. They were defined using the Shore A scale, which measures resistance when a given probe is pressed into the material.

Moreover, a 1-g piece of paraffin, produced in the research laboratory, was used as the control.

Participants used all the products in a randomised order and were blinded to the identity of the product used during each session to minimise bias. The subjects chewed all test products, except for Salivin, which was used by actively sucking. The design of the study is displayed in Figure 1.

**Fig 1 Fig1:**
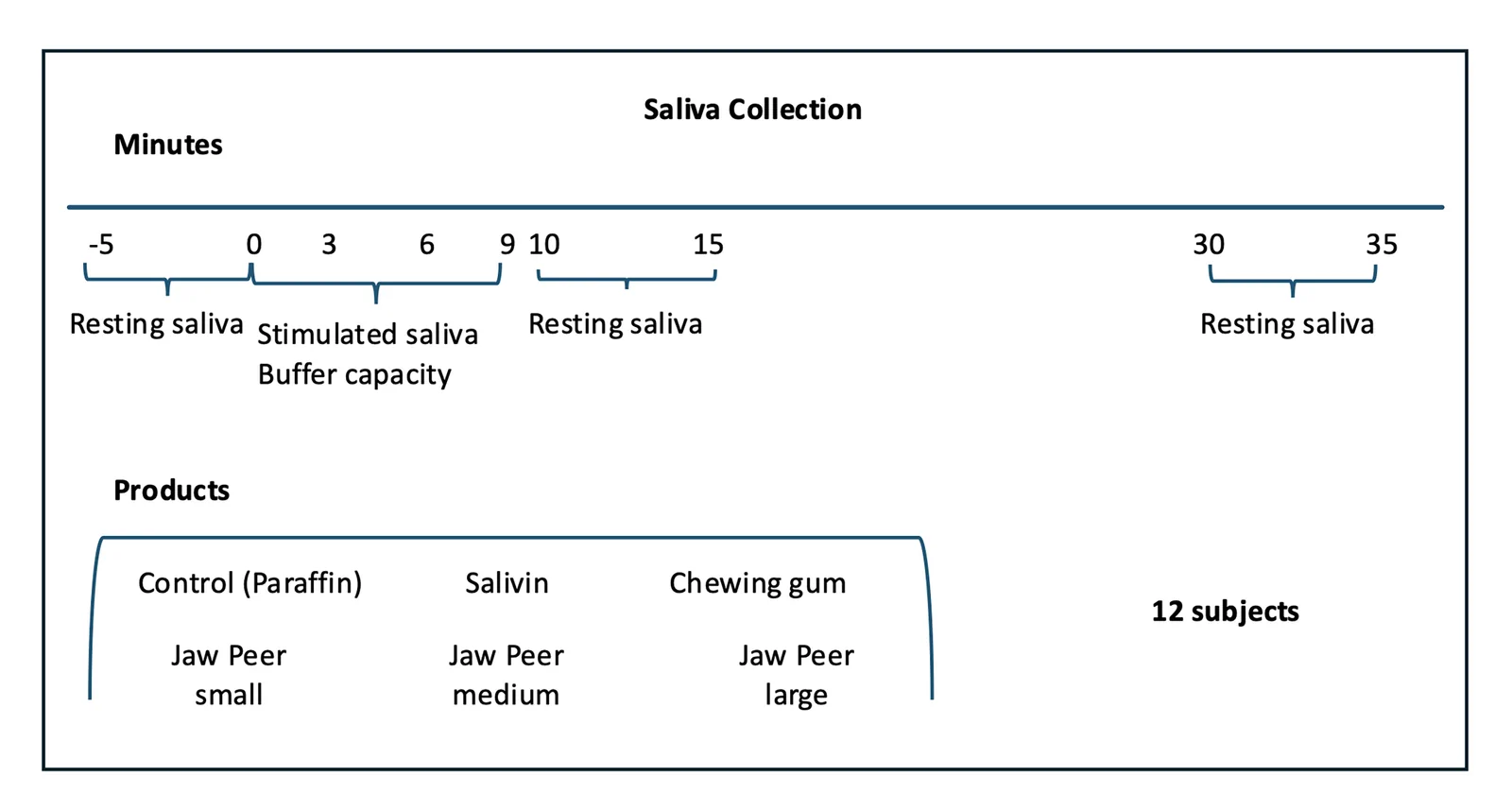
Design and flow-chart of the study.

### Test Procedure

The clinical experiment was conducted over six consecutive days. Each day, participants came to the research laboratory after refraining from eating, drinking, chewing gum, and brushing their teeth for 1 h before testing. They rinsed their mouths with 10 ml of distilled water before each test. Detailed instructions on the proper use of each saliva-stimulating product were provided. Saliva collection occurred at specific time intervals during and after product use. Resting salivary secretion rate, stimulated salivary secretion rate, and buffer capacity were determined at different time points and intervals, as shown in Figure 1. Resting saliva was collected passively for 5 min at baseline (−5 to 0 min), 10 min (10–15 min), and 30 min (30–35 min) after stimulated saliva collection. For stimulated saliva, participants used the products for 9 min and saliva was collected separately at 0–3 min, 4–6 min, and 7–9 min intervals. Saliva collection rates were measured in millilitres per min (ml/min).

Buffer capacity was assessed by transferring 1 ml of stimulated saliva to a beaker containing 3 ml of 0.001 M HCl and 1 drop of octyl alcohol. Air was blown through the sample for 20 min, after which the final pH was determined.

### Statistical Analysis

All saliva and buffer capacity measurements were performed in duplicate and recorded in a custom-prepared Excel ad hoc spreadsheet. After quality control, the data were exported to GraphPad Prism for Mac 10.0 for analysis. Data are presented as as means ± SDs; Fisher’s g for asymmetry was also calculated. Comparisons between products and among time points for the same product were performed using two-way ANOVA followed by Tukey’s post hoc test. Changes in pH (Delta-pH) related to buffering capacity were also evaluated. A significance level of *p* < 0.05 was used.

## Results

The resting saliva measurements did not differ statistically across the three resting salivary flow rates did not differ significantly across the three assessment periods (Table 1). Only in the chewing-gum group did resting saliva decrease from 0.36 to 0.31 ml/min (*p* = 0.02). For stimulated saliva, statistically significant differences were observed, with a higher secretion rate for JAW PEER large compared with paraffin (*p *= 0.02) and Salivin (*p* = 0.01). Salivin further displayed a noteworthy temporal variation, indicating a significant divergence in saliva quantity from the initiation to the conclusion of the experiment (Fig 2). Marginal numerical fluctuations were observed in the buffering capacity of stimulated saliva over the temporal intervals (Fig 3). The highest buffering capacity was observed for saliva collected during the 0–3 min interval, showing a statistically significant difference compared with other collection periods for chewing gum and JAW PEER small and large (*p* < 0.05, *p* < 0.01, or *p* < 0.001). The comparison of the D-pH values provided similar results (data not shown). An additional noteworthy finding was a significantly greater buffering capacity for saliva collected during the 4–6 min interval when Salivin was compared with JAW PEER small (*p* < 0.05). A temporal analysis disclosed a diminishing trend in buffering capacity over time for Salivin and chewing gum, while such a trend remained inconspicuous for paraffin and two of the JAW PEER products.

**Table 1 Table1:** Resting saliva measurements for 5 min: at baseline (−5 to 0 min) before the start of the chewing experiment, after 10 min (10–15 min) and after 30 min (30–35 min) after stimulated saliva collection in the five products plus the paraffin (control). The data are reported as mean (m) and standard deviation (SD) per min

Measurements	Salivin mean ± SD	Chewing gum° mean ± SD	Jaw Peer small mean ± SD	Jaw Peer medium mean ± SD	Jaw Peer large mean ± SD	Control mean ± SD
Baseline (−5 to 0 min)	0.34 ± 0.14	0.36 ± 0.12°	0.31 ± 0.12	0.32 ± 0.06	0.35 ± 0.08	0.31 ± 0.15
10 min (10–15 min)	0.32 ± 0.11	0.37 ± 0.13	0.33 ± 0.19	0.30 ± 0.07	0.33 ± 0.08	0.33 ± 0.10
30 min (30–35 min)	0.35 ± 0.11	0.31 ± 0.10°	0.31 ± 0.14	0.29 ± 0.07	0.30 ± 0.08	0.32 ± 0.15
Two-way ANOVA	*p* = 0.36	*p* = 0.02	*p* = 0.93	*p* = 0.77	*p* = 0.44	*p* = 0.94
Differences among the different measurements and products were assessed using Analysis of Variance (two-way ANOVA). ° within the same column indicate statistically significant differences between time points (*p* < 0.05).						

**Fig 2 Fig2:**
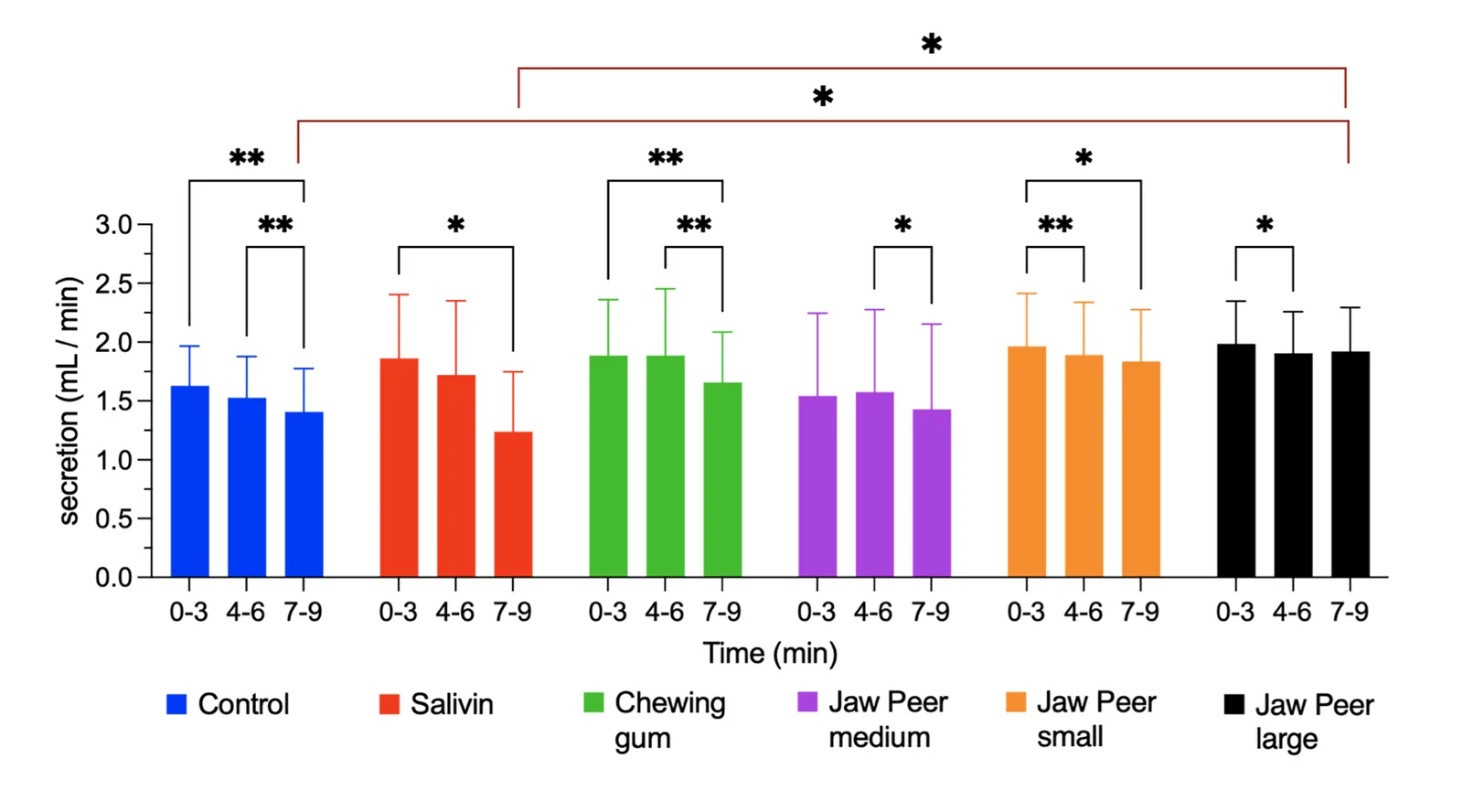
Stimulated salivary secretion during various time intervals expressed in ml/min for the five tested products plus the control/paraffin product. Two-way ANOVA **p* < 0.05; ***p* < 0.01.

**Fig 3 Fig3:**
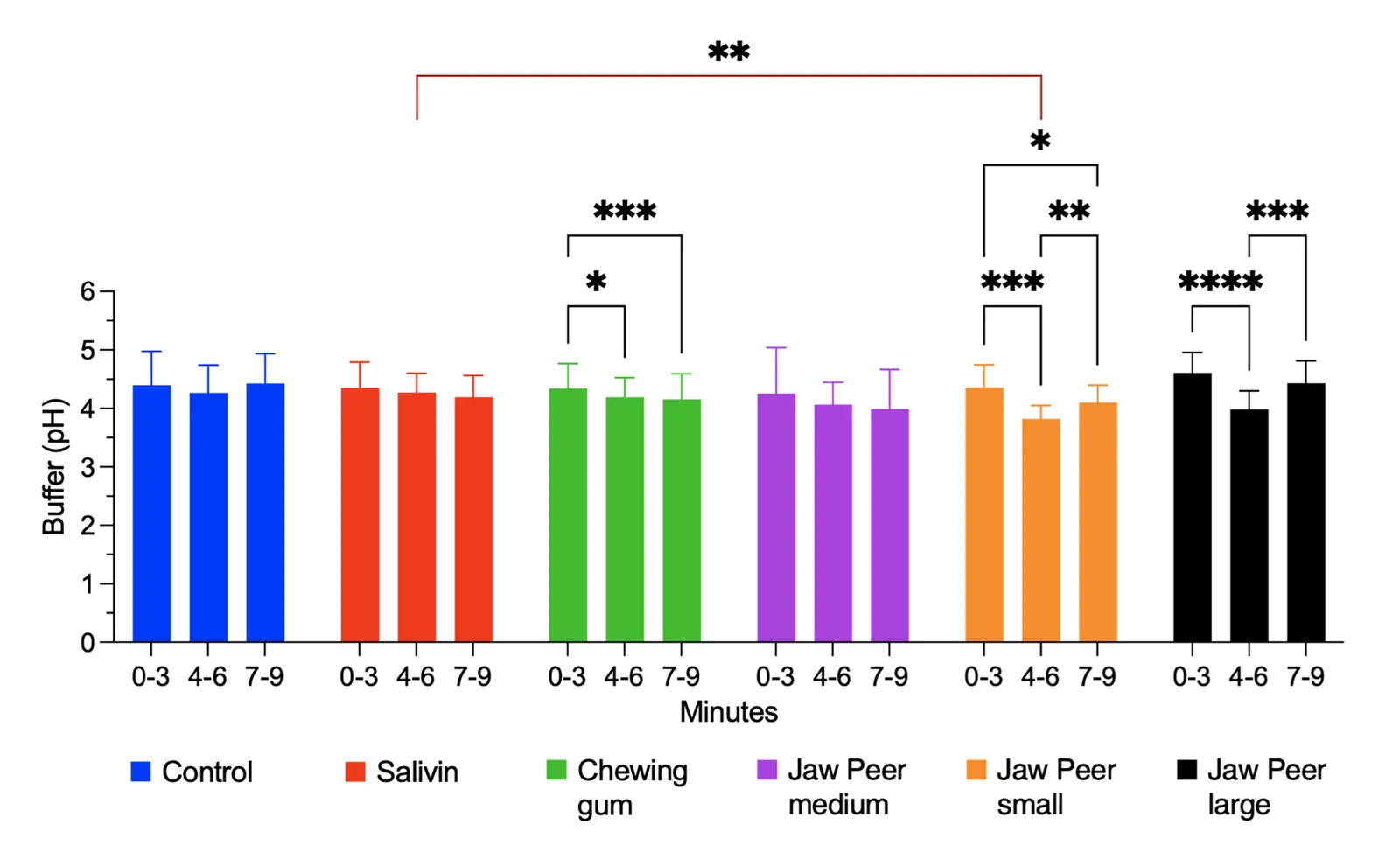
Buffer capacity for saliva collected during different time intervals expressed as final pH for the six tested products. Two-way ANOVA **p* < 0.05; ***p *< 0.01, ****p* < 0.001, *****p* < 0.0001.

## Discussion

The present study aimed to clarify the interplay between saliva-stimulating products, their mode of stimulation, and their effects on salivary flow, buffering capacity, and temporal dynamics. This knowledge may offer useful insights for clinicians managing xerostomia and help them personalise treatment plans to optimise oral health outcomes.

Recent systematic reviews and clinical investigations have further emphasised the importance of individualised xerostomia management and the need for comparative studies assessing functional salivary parameters.^3,13
^ However, limited evidence is available comparing mechanical and gustatory stimulation under standardised experimental conditions, highlighting the relevance of the present pilot study.

The observed divergence in stimulated saliva secretion rates, particularly with the JAW PEER large product, suggests that the mode of stimulation plays a crucial role in salivary gland activation and secretion efficiency. Some products rely mainly on mechanical stimulation, whereas others, such as chewing gum and Salivin, also involve gustatory stimulation. This distinction is important for understanding the multifaceted regulation of saliva production, as taste stimulation can activate additional neural pathways that influence salivary secretion beyond mechanical stimulation alone.^11,13
^ The potential impact of taste suggests a more nuanced approach to product selection, taking into account the individual preferences and responses of patients.

Similarly, a gum’s flavour can influence salivary flow rate (SFR) and pH. The greatest increase in SFR was observed during the first minute of chewing strawberry-flavoured gum. The highest elevation in chewing-stimulated salivary pH was noted with cinnamon- and spearmint-flavoured gums.^8^


Although JAW PEER large produced a high stimulated salivary secretion rate, its effect appeared lower than that of the mint chewing gum. This suggests that mechanical stimulation alone may not be sufficient to achieve maximal salivary flow and that gustatory stimulation may further enhance salivary gland activation.^7,8
^ The combination of mechanical and taste stimuli therefore appears to play an important role in optimising secretion rate. Further investigations into the optimal design features of such products, including size, texture, and shape, may yield insights into the development of more effective interventions for xerostomia.

The temporal analysis of buffering dynamics contributes valuable information about the sustained effectiveness of the saliva-stimulating products. The highest buffering capacity observed during the 0–3 min interval suggests an immediate and robust response to stimulation. This finding may have clinical implications for individuals seeking rapid relief from xerostomia symptoms. Additionally, the observed diminishing trend in buffering capacity over time for certain products, such as Salivin and chewing gum, warrants consideration in long-term management strategies. Understanding the temporal dynamics allows for a more nuanced prescription of these products based on the duration of desired effects.^15^


Furthermore, the significantly augmented buffering capacity for saliva collected during the 4–6 min interval with Salivin compared with JAW PEER small highlights the product-specific variations that clinicians should consider when tailoring treatment plans. The variability in buffering capacity over time may be attributed to the specific formulations and compositions of the products, calling for a deeper exploration of the biochemical mechanisms underlying their buffering properties.^2,12
^


The least pronounced effect was observed with the Jaw Peer Soft product, particularly in terms of stimulated salivary secretion rate, suggests that while it excels in certain aspects, a comprehensive evaluation of a product’s overall efficacy should consider multiple parameters. This nuanced understanding is essential for healthcare professionals to make informed recommendations based on the specific needs and preferences of individual patients.^3,5,10
^


While the present study provides valuable insights into the comparative analysis of saliva-stimulating products, it also acknowledges multiple limitations that warrant consideration. While statistically significant differences were observed in some comparisons, larger-scale studies are needed to confirm present findings and generalise them to broader populations. These limitations highlight the need for further research to validate and deepen our understanding of the complex interactions between saliva-stimulating products, their modes of stimulation, and individual responses.

## Conclusion

This pilot study established that saliva-stimulating products demonstrate varying degrees of effectiveness in enhancing salivary flow rate and buffering capacity over time. Chewing-gum products produced higher stimulated secretion rates, likely due to the combined mechanical and gustatory stimulation they provide. In contrast, Jaw Peer products exhibited more consistent buffering capacity, though they did not outperform the control (paraffin) consistently. The limited differences in buffering capacity compared with the paraffin control indicate that mechanical stimulation alone already provides substantial buffering support, and that additional product-specific benefits may be moderate. Clinically, products combining taste and mechanical stimulation may be preferred when rapid salivary flow enhancement is desired, whereas mechanically based devices may offer more sustained but moderate effects. Given the small sample size and pilot design, larger studies in xerostomic patients are required before definitive clinical recommendations can be established.

### Acknowledgements

We sincerely thank Mohaned Albovad and Zena Albovad for their valuable assistance with data collection. We also extend our gratitude to JAW PEER AB, Vallentuna, Sweden, for providing the products used in the study.

## References
